# Efficiency of the Sensory Mapping Score for Hansens’s disease diagnosis and follow-up: a functional cure criterion?

**DOI:** 10.3389/fmed.2025.1685347

**Published:** 2025-11-20

**Authors:** Marco Andrey Cipriani Frade, Gustavo Sartori Albertino, João Vitor da Silva Sabino, Filipe Rocha Lima

**Affiliations:** 1 Dermatology Division, Department of Internal Medicine, Ribeirão Preto Medical School, University of São Paulo, São Paulo, Brazil; 2 National Referral Center for Sanitary Dermatology and H.D., Clinical Hospital, Ribeirão Preto Medical School, University of São Paulo, São Paulo, Brazil; 3 Laboratory for Skin Studies and Alternative Models, Ribeirão Preto Medical School, University of São Paulo, São Paulo, Brazil; 4 University of Ribeirão Preto (UNAERP), São Paulo, Brazil; 5 Department of Biochemistry and Immunology, Ribeirão Preto Medical School, University of São Paulo, São Paulo, Brazil

**Keywords:** Hansen’s disease, leprosy, Semmes-Weinstein Monofilaments, Sensitive Mapping Score, cutaneous lesions, functional recovery

## Abstract

**Introduction:**

Current operational criteria for Hansen’s disease (HD), which are primarily based on lesion count and fixed-duration multidrug therapy, tend to overlook the neurological spectrum of the disease and fail to define cure in terms of functional recovery. The objective was to develop and validate a Sensitive Mapping Score using Semmes-Weinstein Monofilaments to assess and monitor cutaneous sensory impairment in HD lesions during treatment with the RIMOXCLAMIN regimen.

**Methods:**

A prospective cohort of 40 patients was followed over a 12-month period. Tactile sensitivity was evaluated in the hands, feet, and cutaneous lesions at 3-month intervals using a standardized SWM mapping system. Color-coded responses were converted into a numerical score reflecting the degree of sensory impairment. Clinical, neurological, and laboratory parameters were assessed in parallel.

**Results:**

Although only 32.5% of patients met the multibacillary classification based on lesion count, 100% exhibited altered tactile sensation and 80% presented with nerve thickening. The SMS demonstrated early sensitivity to neurological improvement, with significant changes observed by the third month (*p* = 0.0156). By 12 months, 74.3% of lesions had achieved complete sensory recovery. The progression of SMS scores paralleled reductions in pain levels, nerve palpation abnormalities, and physical disability grades. No linear correlation was found with standard hand and feet scores, reinforcing the SMS’s complementary value in capturing localized sensory recovery.

**Conclusion:**

The SMS is a low-cost, accessible, and objective tool for neurological monitoring in HD. It quantifies functional recovery in cutaneous lesions, offering a clinically meaningful alternative to the concept of an “administrative cure.” Its adoption could guide individualized treatment durations, enhance therapeutic decision-making, and promote a shift toward function-based definitions of cure in HD management.

## 1 Introduction

Hansen’s disease (HD) is a chronic infectious disease with an insidious course, caused by *Mycobacterium leprae* and *M. lepromatosis*. Its primary tropism involves superficial cutaneous nerves and peripheral nerve trunks, although it may also affect the eyes and, in more advanced stages, internal organs (2). It is one of the leading infectious causes of peripheral neuropathy worldwide, with significant potential to cause sequelae and physical disability (3).

According to the World Health Organization (WHO), HD is classified as a neglected disease due to its low prioritization in public health agendas, despite being a relevant health problem that causes social stigma and substantial physical limitations in affected individuals (4). This marginalization is associated with social and economic factors that increase transmission risk and hinder early diagnosis and adequate treatment, particularly in high-incidence regions such as Brazil and other countries where living conditions facilitate disease spread (4). Globally, more than 200,000 cases have been detected annually, with Brazil ranking second in reported cases and struggling to meet the elimination target of fewer than 10 new cases per 100,000 inhabitants (5). In 2024, Brazil reported approximately 22,129 new HD cases, corresponding to a detection rate of 10.43 per 100,000 inhabitants, indicating a very high endemic status (6). Brazil, India, and Indonesia together account for approximately 80% of all reported cases worldwide (7). However, these figures are likely underestimated, as the country has yet to return to pre-pandemic levels, which in 2019 recorded 27,864 new cases and a rate of 13.23 per 100,000 inhabitants, classified as high endemicity (8). Within the Americas, Brazil continues to account for over 90% of all new reported cases, highlighting a highly unequal and concerning regional distribution (9).

Per WHO guidelines, the diagnosis of HD is based on the presence of at least one of three cardinal signs: loss of sensation in hypopigmented or erythematous skin lesions; thickening of peripheral nerves associated with sensory loss and/or muscle weakness; or detection of acid-fast bacilli in slit-skin smear microscopy. Early diagnosis of paucibacillary (PB) forms and mild borderline cases remains challenging due to the low accuracy of serological tests such as enzyme-linked immunosorbent assay (ELISA) or lateral flow assays. Polymerase chain reaction (PCR)-based techniques, while more sensitive and specific, still lack standardization and widespread accessibility, and less invasive alternatives such as urine or blood sampling present limited sensitivity (10).

Neuropathy in HD is a major clinical manifestation resulting from *M. leprae* infection of peripheral nerves, in addition to host inflammatory and immunological responses. The literature documents that HD-associated neuropathy severely impacts patients’ health and quality of life, contributing to hypoesthesia or anesthesia, muscle weakness, paralysis, and disabling deformities of the extremities, particularly the fingers and toes due to involvement of specific peripheral nerves including the ulnar, radial, and median nerves in the upper limbs, and the common fibular, peroneal, and posterior tibial nerves in the lower limbs (11, 12). Antigenic mimicry by *M. leprae* may help the bacterium evade immunity, particularly in lepromatous HD, and trigger autoimmune responses, as antibodies can cross-react with human nerve and skin cells, contributing to nerve damage (13).

Historically, tactile sensitivity has been used in neurological assessments, such as a sensitivity test for HD since 1969 (14), and Von Frey’s method, designed in 1896, was later used to measure pressure sensitivity in the hand with Weinstein-Semmes esthesiometer in 1978 (15). Evidence indicates that neurosensory assessment should be prioritized in HD diagnosis, as neural manifestations often precede or coexist with dermatological findings (16, 17). Hypochromic macular lesions associated with sensory dysfunction are a common finding regardless of clinical spectrum. The use of Semmes-Weinstein Monofilaments (SWM) esthesiometry has emerged as an objective strategy for quantifying sensory loss, revealing characteristic patterns such as preservation of sensitivity in surrounding areas (the “island” pattern) (18, 19).

Standardization of the 0.07 gram-force (gf) green monofilament as the threshold for normal tactile sensation has demonstrated high diagnostic accuracy, with 97.9% sensitivity and 98.6% specificity in identifying macular abnormalities. Moreover, measurement of the internal-to-peripheral sensory index difference (Δ > 0.34) has been established as a robust marker, showing 100% sensitivity and 96.5% specificity for HD. These findings highlight the potential of sensory evaluation not only for early diagnosis but also for longitudinal monitoring of therapeutic response (20). The SWM test is a standardized, low-cost, and highly accurate method for evaluating cutaneous tactile sensation across peripheral nerve territories, and the Brazilian Ministry of Health recommends a six-filament esthesiometry kit for HD monitoring. This ensures technical fidelity aligned with historical validation standards, including studies from the HD Center in Carville.

Treatment of HD has historically relied on the multidrug therapy (MDT) regimen standardized by the WHO (MDT/WHO), composed of rifampicin, dapsone, and clofazimine. However, new therapeutic approaches have been investigated to enhance bactericidal efficacy and reduce treatment duration. Among these alternatives, the RIMOXCLAMIN regimen, comprising rifampicin, moxifloxacin, clarithromycin, and minocycline, has demonstrated faster restoration of tactile sensitivity in the hands and feet, as assessed by SWM during the third month of follow-up. RIMOXCLAMIN outperformed the conventional MDT/WHO regimen, also showing favorable clinical outcomes in pain reduction, peripheral nerve palpation findings, physical disability grade, and adverse event profile, underscoring its potential to optimize HD control and improve cure criteria (21).

The incorporation of standardized techniques such as graphic and photographic sensitive mapping enhances objective documentation of disease diagnosis, progression, and therapeutic efficacy. Systematic adoption of these methods in clinical practice strengthens an evidence-based approach, enabling early interventions that minimize deformities and improve patient prognosis (22). In light of findings regarding tactile sensitivity assessment using SWM on macules and/or pathognomonic areas for HD diagnosis, this article aims to validate the effectiveness of the Sensitive Mapping Score (SMS) applied to HD skin lesions. This tool may become a strategic and indispensable asset in addressing diagnostic and therapeutic follow-up challenges in HD management.

## 2 Patients and methods

### 2.1 Study design and casuistic

This is a bidirectional study aimed at standardizing the SMS as a clinical classification tool for the diagnosis and monitoring of HD. Data were obtained from a photographic database comprising dermatological lesion images taken both at the time of diagnosis and during clinical and therapeutic follow-up. These included patients treated at the outpatient clinics of the National Referral Center in Sanitary Dermatology, Clinical Hospital of Ribeirão Preto Medical School, University of São Paulo, Brazil, as well as patients referred from the Civil Clinic, a private service affiliated with the hospital.

All images included in the analysis were selected based on quality and diagnostic representativeness by experienced dermatologists and hansenologists. From a larger cohort of over 100 patients, only those with complete photographic records at baseline and at 3-, 6-, and 12-months during treatment were included, ensuring standardized quarterly follow-up until discharge. Patients lacking any of these scheduled evaluations were excluded from the study. The database comprises both untreated and treated patients, enabling comparative assessments throughout the therapeutic course. This dual temporal design supported the inclusion of cases for score calibration and prospective validation, thereby strengthening the applicability of the proposed sensitivity-mapping tool in both diagnostic and follow-up contexts.

### 2.2 Neurodermatological evaluation

Patients (*N* = 40) were followed by the hansenologist at least quarterly, with systematic evaluations of neurological symptoms and cutaneous signs to monitor disease progression or improvement. The strength symptom (weakness) was defined based on complaints and medical record reviews of standard strength assessments, while other symptoms were identified through anamnesis. Sensory mapping assessments were performed at diagnosis, after 3 and 6 months, and at the end of treatment.

Tactile sensitivity was evaluated at seven standardized points on each hand and 11 points on each foot using SWM, 38 mm in length and calibrated to exert a defined axial force ranging from 0.07 to 300 gram-force (gf) (10, 21). These filaments are color-coded green (0.07 gf), blue (0.2 gf), violet (2.0 gf), red (4.0 gf), orange (10.0 gf), pink (300 gf), and black (> 300 gf) to enable semi-quantitative assessment of sensory loss. The green monofilament (0.07 gf), identified by Semmes and Weinstein with the logarithmic code “2.83,” initiates buckling with approximately 0.068–0.07 gf and is widely accepted as the tactile threshold of normality for the hands and trunk (21, 22). For plantar regions, the blue filament (0.2 gf) is considered the normal threshold due to thicker skin (10, 23). The monofilaments used in this study were manufactured by SORRI-BAURU, Brazil, following the original specifications by Semmes and Weinstein, using identical nylon type and extrusion diameter, and according to the Brazilian Ministry of Health guidelines. Similarly, SWMs ranging from 0.07 to 300 gf were used to assess tactile sensitivity on hypochromic macules. As described by Frade et al. (10, 22), perception of the green monofilament (0.07 gf) in these areas was classified as a normal tactile threshold. The test progression followed a stepwise approach, with the color corresponding to the smallest monofilament perceived by the patient being recorded.

The esthesiometry findings from cutaneous lesions were individually documented, organized, and interpreted alongside the SMS described in Table 1, proposed by Frade since 2022. This integrated evaluation contributed to more precise stratification of skin involvement and provided a reproducible tool to longitudinally monitor the patient’s clinical course. The analyses were performed by comparing and correlating the esthesiometric findings and the SMS applied to the results of esthesiometry of hands and feet and applied to the lesions (average score of each patient): (a) aSWM: Sum of altered Semmes-Weinstein points; (b) aSWM-H: Number of Semmes-Weinstein points altered on hands; (c) aSWM-F: Number of Semmes-Weinstein points altered on feet; (d) SMS-H: Sensitive Mapping Score applied on hands by SWM esthesiometry; (e) SMS-F: Sensitive Mapping Score applied on feet by SWM esthesiometry; (f) SMS-L: Sensitive Mapping Score on lesions.

**TABLE 1:** Color-based tactile sensitivity score for skin lesions using SWM proposed by Frade et al. (22).

Color (gf)	Scoring system based on color-response
G—Green (0.07 g)	0
B—Blue (0.02 g)	1
V—Violet (2 g)	2
R—Red (4 g)	4
O—Orange (10 g)	5
P—Pink (300 g)	10
Black—Black (> 300)	20
Σ	42

### 2.3 Definition of H.D. cases and classification

As described by Lima et al. (5) all patients were diagnosed through clinical evaluation performed by experienced leprologists, following the Brazilian Ministry of Health and WHO guidelines using the recommended cardinal signs (2, 3, 20, 24–26). Auxiliary diagnostic tests included assessment of tactile sensation with SWM, bilateral high-resolution ultrasound of the peripheral nerves (USG-PN), electroneuromyography (ENMG), and other complementary exams such as ELISA anti-phenolic glycolipid-I (aPGL-I) serology, qPCR-RLEP, and bacilloscopy, as recorded in the medical records (10, 20, 25–30).

Patients were classified according to the adapted Madrid (Congress of Madrid 1953) and the Indian Association of Leprology (IAL 1982) classifications as follows: indeterminate (I), polar tuberculoid (TT), borderline-borderline (BB), borderline lepromatous (BL), polar lepromatous-lepromatous (LL), and pure neural HD (PNL) (31–33); and as paucibacillary (PB) (I and TT clinical forms) or multibacillary (MB) (BB, BL, LL, and PNL forms) according to WHO operational criteria. According to Frade et al. (20), patients presenting hypo or depigmented macules with altered sensation and neurological findings were classified as borderline/MB. The use of techniques as peripheral nerve ultrasound enables the early detection of neurological changes, which can also be observed in the initial stages of the disease (34, 35).

### 2.4 Statistical analysis

All data were compiled using Microsoft Excel 2023 and subsequently analyzed with GraphPad Prism version 10.0 (GraphPad Inc., La Jolla, CA, United States). For comparisons of tactile sensitivity responses across different time points during patient follow-up, the non-parametric Wilcoxon matched-pairs signed-rank test was applied. Correlations among continuous or ordinal variables were assessed using Spearman’s rank correlation coefficient (ρ – rho). The strength of correlations was classified as absent (< 0.3), weak (approximately 0.3–0.5), moderate (0.5–0.7), or strong (> 0.7), according to established methodological guidelines (36). Continuous variables were described using medians and interquartile ranges (IQR), while categorical variables were presented as absolute frequencies and percentages. The significance level was set at 95% (alpha = 0.05), with *p*-values considered significant when ≤0.05. Results were expressed with corresponding 95% confidence intervals (95% CI). For contingency analyses involving dichotomous categorical variables (e.g., presence or absence of sensory perception by color threshold), Fisher’s exact test was used when expected frequencies were low. The analysis framework was designed to assess intra-individual changes over time in a paired manner, reinforcing sensitivity score progression as a longitudinal clinical endpoint.

## 3 Results

### 3.1 Demographic, clinical and laboratory characterization of patients

Demographic and clinical data of the individuals prior to treatment initiation are presented in Table 2. Regarding sex distribution, 67.5% of the sample were male, with a median age of 54.5 years. Clinically, all patients (100%) were classified as borderline. The median symptom duration before diagnosis was 30 months. Regarding the endogenous histamine test, 77.5% of patients exhibited altered reactivity, indicating frequent impairment of cutaneous neurovascular responses. In terms of cutaneous involvement, 32.5% of individuals presented with more than five skin lesions at baseline. Concerning sensitivity alterations, 55% of the cohort showed neural signs and symptoms involving pain, light touch, and thermal sensitivity. When analyzed individually, the most prevalent tactile abnormality was loss of light touch (100%), followed by impaired thermal sensation (65%) and pain perception (60%).

**TABLE 2:** Baseline demographic and clinical characteristics of patients with Hansen’s disease.

Features		*N* = 40	
		*n*	%
Gender	Men	27	67.5
	Women	13	32.5
Age (years)	Median (IQR)	54.5 (45–67)	
Clinical classifications	B^1^	40	100
Symptoms duration (in months)	Median (IQR)	30 (12–54)	
**Skin assessment**			
Endogenous histamine test	Normal	9	22.5
	Altered	31	77.5
Number of skin lesions	0	–	–
	1	7	17.5
	2–5	20	50
	> 5	13	32.5
Sensory alteration	Tactile	40	100
	Thermal	26	65
	Painful	24	60
	Tactile and thermal	25	62.5
	Tactile and painful	24	60
	Thermal and painful	22	55
	Tactile, thermal, and painful	22	55

IQR, Interquartile Range.

^1^B, Borderline. Dashes (–) indicate null value.

Data on neural impairment and Physical Disability Grade (PDG) at baseline and after 3, 6, and 12 months of treatment are summarized in Table 3. Regarding nerve palpation, a marked reduction in the frequency of nerve thickening was observed, decreasing from 80% at baseline to 32.5% at 12 months (*p* < 0.0001). Similarly, the proportion of patients reporting pain on nerve palpation declined from 45% at baseline to 5% after 12 months (*p* < 0.0001).

**TABLE 3:** Nerve palpation findings and physical disability grade (PDG) at baseline and during treatment follow-up.

Follow-up component		Initial		3rd MO		6th MO		12th MO		*P*-value^1^ (follow-up)				
		n	%	n	%	n	%	n	%	Initial to 3rd MO	3rd to 6th MO	Initial to 6th MO	6th to 12th MO	Initial to 12th MO
Palpation	≥1 Thickened	32	80	24	60	22	55	13	32.5	0.0002	0.006	< 0.0001	0.003	< 0.0001
	≥1 Painful	18	45	14	35	10	25	2	5	0.035	0.07	0.001	0.06	< 0.0001
PDG	PDG 0	6	15	15	37.5	25	62.5	31	77.5	**0.002**	**0.002**	** < 0.0001**	**0.03**	** < 0.0001**
	PDG 1	23	57.5	17	42.5	12	30	7	17.5					
	PDG 2	11	27.5	8	20	3	7.5	2	5					

PDG, Physical Disability Grade.

^1^Wilcoxon signed-rank test. Values in bold indicate statistically significant *p*-values.

PDG showed progressive improvement over time. The proportion of patients classified as PDG 0 (no physical disability) increased significantly from 15% at baseline to 37.5% at 3 months (*p* = 0.0018), 62.5% at 6 months (*p* < 0.0001), and 77.5% at 12 months (*p* < 0.0001), indicating substantial recovery of function and prevention of long-term sequelae.

Neurological symptom data collected throughout follow-up are presented in Table 4. A significant reduction in the frequency of tingling was observed, decreasing from 80% at baseline to 55% after 3 months of treatment (*p* = 0.002). Similarly, muscle cramps decreased from 67.5 to 45% in the same period (*p* = 0.003), and weakness declined from 65 to 37.5% (*p* = 0.007). Numbness showed a significant reduction only after 6 months of treatment, decreasing from 95 to 37.5% (*p* < 0.0001). Pain-related symptoms (e.g., burning, prickling, stinging) also declined significantly, from 75% at baseline to 35% at 6 months (*p* = 0.0001). Between the 6 and 12th months of treatment, all neurological symptoms continued to improve significantly (*p* < 0.05), except for muscle weakness, which did not reach statistical significance in this interval (*p* = 0.07).

**TABLE 4:** Frequency of neurological symptoms (numbness, tingling, cramps, weakness, neuropathic pain) during follow-up.

Follow-up symptom	Initial		3rd MO		6th MO		12th MO		*P*-value^1^ (follow-up)				
	n	%	n	%	n	%	n	%	Initial to 3rd MO	3rd to 6th MO	Initial to 6th MO	6th to 12th MO	Initial to 12th MO
Numbness	38	95.0	33	82.5	15	37.5	7	17.5	0.06	**0.0001**	** < 0.0001**	**0.0002**	** < 0.0001**
Tingling	32	80.0	22	55.0	15	37.5	2	5.0	**0.002**	0.06	** < 0.0001**	**0.001**	** < 0.0001**
Cramps	27	67.5	18	45.0	10	25.0	3	7.5	**0.003**	**0.0215**	** < 0.0001**	**0.039**	** < 0.0001**
Pain^2^	30	75.0	24	60.0	14	35.0	6	15.0	0.07	**0.0063**	**0.0001**	**0.039**	** < 0.0001**
Weakness	26	65.0	15	37.5	9	22.5	3	7.5	**0.007**	0.07	** < 0.0001**	0.07	** < 0.0001**

^1^ Wilcoxon signed-rank test.

^2^ Burning, Stinging, Needles. Values in bold indicate statistically significant *p*-values.

Pain intensity outcomes assessed over time are detailed in Table 5. At baseline, only 20% of patients reported pain scores below 3 on the Numerical Rating Scale (NRS). After 3 months of treatment, this proportion increased significantly to 50% (*p* = 0.0001). Further improvement was observed at subsequent follow-ups: at 6 months, 85% of patients reported pain scores below 3 (*p* < 0.0001), and by 12 months, this number reached 95% (*p* = 0.0002), indicating a consistent and substantial reduction in pain perception throughout the treatment period.

**TABLE 5:** Distribution of patients according to pain scores at baseline and during follow-up.

Follow-up pain scale	Initial		3rd MO		6th MO		12th MO		*P*-value^1^ (follow-up)				
									Initial to 3rd MO	3rd to 6th MO	Initial to 6th MO	6th to 12th MO	Initial to 12th MO
0–2	8	20	20	50	34	85	38	95	**0.0001**	** < 0.0001**	**<0.0001**	**0.0002**	** < 0.0001**
3–5	19	47.5	13	32.5	4	10	2	5					
> 5	13	32.5	7	17.5	2	5	0	–					

^1^Wilcoxon signed-rank test. Dashes (–) indicate null value. Values in bold indicate statistically significant *p*-values.

Complementary diagnostic test data obtained at any time during follow-up (diagnosis or treatment) are summarized in Table 6. Regarding molecular testing, 37 patients underwent RLEP-PCR, with positivity detected in only 9 cases (24.3%). For serological analysis, 38 samples were tested by ELISA aPGL-I, with only 3 positive results (7.9%). Bacilloscopy of the slit-skin smear was performed in 11 patients, with a positivity rate of 36.4% (4/11). All patients underwent at least one laboratory diagnostic test. Of the total cohort, 28 patients (70%) tested negative in all three laboratory methods (RLEP-PCR, ELISA, and bacilloscopy).

**TABLE 6:** Results of complementary diagnostic tests performed during follow-up.

Complementary exams		*N* = 40	
		*n*	%
RLEP-PCR	Exams performed	37	92.5
	Positive	9	24.3
	Negative	25	67.6
	Inconclusive	3	8.1
ELISA aPGL-I	Exams performed	38	95
	Positive	3	7.9
	Median (range)	1.3 (1.2–1.3)	
	Negative	35	92.1
Bacilloscopy	Exams performed	11	27.5
	Positive	4	36.4
	Median (range)	1 (1–2)	
	Negative	7	63.6
Laboratorials^1^	At least 1 performed	40	100
	= 1 positive test	8	20
	= 2 positive tests	4	10
	= 3 positive tests	0	–
USG-PN^2^	Exams performed	40	100
	Normal	0	–
	MNPM^3^	40	100
	Other pathologies	0	–
ENMG^4^	Exams performed	37	92.5
	Normal	1	2.7
	MNPM^3^	34	91.9
	Other pathologies	2	5.4
USG-PN^2^ and/or ENMG^4^	Both normal	0	–
	MNPM^3^ at 1 exam	6	15
	MNPM^3^ at both^5^	34	85

^1^ Laboratory: RLEP-PCR, ELISA aPGL-I and cutaneous bacilloscopy.

^2^ USG-PN: bilateral high-resolution ultrasound of the peripheral nerves.

^3^ MNPM: Multiple mononeuropathy.

^4^ ENMG: Electroneuromyography.

^5^ Both: USG-PN and ENMG;

^6^ BI: bacilloscopy index. Dashes (–) indicate null value.

Regarding neurological imaging, all patients (100%) underwent peripheral nerve ultrasonography, which confirmed the presence of multiple mononeuropathy (MPNM) in all cases. ENMG was performed in 37 patients, with MPNM identified in 34 (91.9%) of them. When considering both USG-PN and ENMG, 85% of patients exhibited abnormalities consistent with MPNM in both modalities.

### 3.2 Analysis of esthesiometric application and sensitive mapping

Tactile sensitivity evaluations of the hands using SWM during follow-up are detailed in Table 7. While no statistical differences were found in the proportion of altered tactile sites when comparing consecutive timepoints (*p* > 0.05), a significant reduction was observed between baseline and the 6th month of treatment (*p* = 0.016). Importantly, when the SMS was applied, a cumulative score reflecting the number and degree of altered tactile points. A statistically significant improvement was already detectable by the third month (*p* = 0.016). No further significant changes were observed after the 6th month, indicating early stabilization of sensory recovery.

**TABLE 7:** Evolution of altered tactile points and sensitivity score in the hands during treatment.

Follow-up SWM		Initial	3rd MO	6th MO	12th MO	*P*-value^1^ (follow-up)				
						Initial to 3rd MO	3rd to 6th MO	Initial to 6th MO	6th to 12th MO	Initial to 12th MO
% of altered points	Normal sensitivity	95.54	97.68	98.57	99.64	0.06	0.25	**0.016**	1	**0.016**
	Blue (0.20 gf)	3.04	1.43	1.07	0.36					
	Violet (2.0 gf)	0.89	0.54	0.36	–					
	Red (4.0 gf)	0.36	0.36	–	–					
	Orange (10.0 gf)	0.18	–	–	–					
	Pink (300 gf)	–	–	–	–					
	Black (> 300 gf)	–	–	–	–					
Sensitive mapping score (SMS)	Normal sensitivity (score = 0)	33	36	39	39	**0.016**	0.25	**0.016**	1	**0.016**
	Median (IQR)	0 (0–0)	0 (0–0)	0 (0–0)	0 (0–0)					
	Min–Max	0–13	0–10	0–10	0–2					

SWM, Semmes-Weinstein Monofilaments.

^1^Wilcoxon signed-rank test. Dashes (–) indicate null value. Values in bold indicate statistically significant *p*-values.

Tactile sensitivity assessments of the feet using SWM are summarized in Table 8. A statistically significant reduction was observed both in the number of altered points and in their corresponding SMSs after 3 months of treatment (*p* < 0.0001 for both comparisons). Between the 3rd and 6th months of treatment, no significant changes were detected in either parameter (*p* > 0.05). Nonetheless, both measures showed further significant improvement from the 6th month to the end of treatment, with *p* = 0.019 for the number of altered points and *p* = 0.035 for the score. These results suggest a biphasic recovery pattern in foot sensitivity, with early gains followed by stabilization and subsequent additional improvement toward the treatment endpoint.

**TABLE 8:** Evolution of altered tactile points and sensitivity score in the feet during treatment.

Follow-up SWM		Initial	3rd MO	6th MO	12th MO	*P*-value^1^ (follow-up)				
						Initial to 3rd MO	3rd to 6th MO	Initial to 6th MO	6th to 12th MO	Initial to 12th MO
% Of altered points	Normal sensitivity	55.34	63.64	73.86	85.91	** < 0.0001**	0.38	** < 0.0001**	**0.019**	** < 0.0001**
	Blue (0.20 gf)	25.45	25.34	16.25	9.09					
	Violet (2.0 gf)	11.93	7.73	7.27	3.75					
	Red (4.0 gf)	3.75	1.82	1.36	0.68					
	Orange (10.0 gf)	1.48	1.25	1.14	0.57					
	Pink (300 gf)	1.25	0.11	0.11	–					
	Black (> 300 gf)	0.80	0.11	–	–					
Sensitive mapping score (SMS)	Normal sensitivity (score = 0)	6	19	23	30	** < 0.0001**	0.07	** < 0.0001**	**0.035**	** < 0.0001**
	Median (IQR)	5 (2–14)	1 (0–5.2)	0 (0–4)	0 (0–0.5)					
	Min–max	0–158	0–42	0–45	0–24					

SWM, Semmes-Weinstein Monofilaments.

^1^Wilcoxon signed-rank test. Dashes (–) indicate null value. Values in bold indicate statistically significant *p*-values.

Data regarding the evaluation of cutaneous lesions using SWM are presented in Table 9. At baseline, no patients demonstrated preserved tactile sensitivity within skin lesions i.e., all assessed areas showed some degree of sensory impairment. After 3 months of treatment, 20.3% of the lesions exhibited normal tactile thresholds (*p* < 0.0001), and no lesion areas remained insensitive to the highest-pressure monofilament (300 gf). At the 6-month follow-up, 46% of the lesions had returned to normal sensitivity (*p* < 0.0001). By the end of treatment, 74.3% of lesion sites presented normal responses to SWM testing (*p* < 0.0001), and no areas required a monofilament ≥ 2.0 gf to elicit a response. When analyzing the mean sensory score per patient based on lesion evaluations, 3 individuals (7.5%) had fully normalized responses after 3 months (*p* < 0.0001). This number increased to 10 patients (25%) at 6 months (*p* < 0.0001), and 21 (52.5%) patients exhibited normal neurodermatological responses at the end of treatment (*p* < 0.0001).

**TABLE 9:** Evolution of tactile sensitivity in skin lesions (*N* = 74) and mean lesion score per patient (*N* = 40) during treatment.

SWM	Body region	Lesions (*N* = 74)				Patients (*n* = 40)			
	*P*-value^1^ (in comparison to the previous assessment)	–	< 0.0001	< 0.0001	< 0.0001	–	< 0.0001	0.0003	< 0.0001
	Time period	Initial	3rd MO	6th MO	12th MO	Initial	3rd MO	6th MO	12th MO
% Of altered points	Normal sensitivity	–	20.27	45.95	74.32	–	7.5	25	55
	Blue (0.20 gf)	95.95	79.73	54.05	25.68	95	92.5	75	45
	Violet (2.0 gf)	89.19	62.16	29.73	5.41	95	77.5	52.5	10
	Red (4.0 gf)	50.00	18.92	12.16	–	75	30	22.5	–
	Orange (10.0 gf)	54.05	12.16	6.76	–	67.5	20	12.5	–
	Pink (300 g f)	12.16	2.70	1.35	–	17.5	5	2.5	–
	Black (> 300 gf)	4.05	–	–	–	7.5	–	–	–
Sensitive mapping score (SMS)	Normal sensitivity (score = 0)	–	20.27	45.95	74.32	–	7.5	25	55
	Median (IQR)	8 (3–12)	3 (1–3)	1 (0–3)	0 (0–1)	7.6 (5.9–12)	3 (1.25–3.8)	1 (0.25–3)	0 (0–0.75)
	Min–max	1–42	0–22	0–22	0–4	1–42	0–22	0–12	0–3

SWM, Semmes-Weinstein Monofilaments.

^1^Wilcoxon matched-pair *t*-test. Dashes (–) indicate null value.

### 3.3 SMS performance in diagnosis and follow-up

**FIGURE 1:**
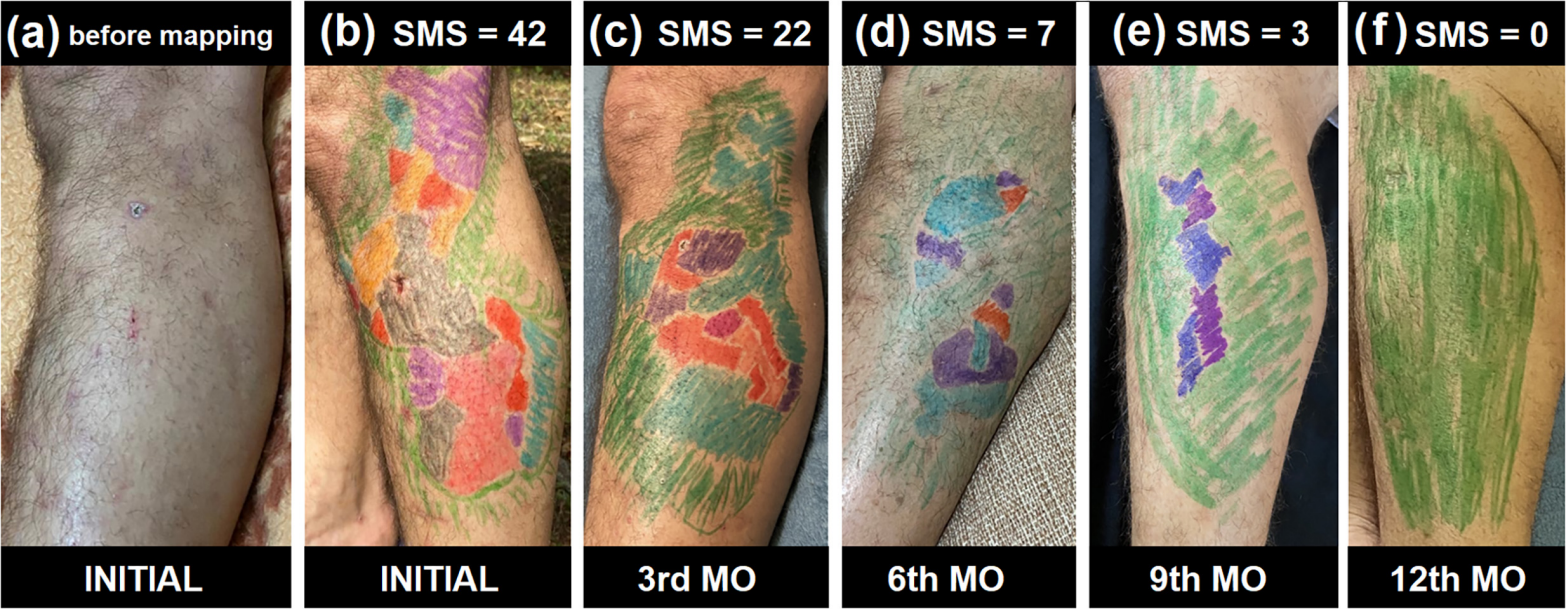
Temporal evolution of cutaneous sensitivity scores during treatment. **(a–f)** Illustrate the progressive recovery of tactile sensitivity in a representative patient included in this study (1 of 40 cases), based on serial Semmes-Weinstein Monofilament (SWM) mappings over 12 months during treatment. **(a)** The clinical appearance of the skin before any mapping showing a large hypochromic patch with thinning hair. **(b)** Presents the baseline sensitivity map with a Sensitive Mapping Score (SMS) of 42. **(c–e)** Correspond to the third, sixth, and ninth months of treatment, with respective SMS values of 22, 7, and 3. **(f)** Depicts the final evaluation at the twelfth month (discharge), demonstrating full sensory recovery with SMS = 0. The color-coded overlays represent the degree of tactile impairment, with gradual replacement by green areas indicating normalization of sensation over time. Color code from the SWM evaluation: GREEN (0.07 g-force); Blue (0.02 g-force); Violet (2 g-force); Red (4 g-force); Orange (10 g-force); Pink (300 g-force); Black (no tactile sensation).

This improvement trajectory is exemplified in Figure 1, which presents sequential SWM mappings from a representative patient (1 of 40) across six timepoints. The before treatment mapping (SMS = 42) shows widespread sensory loss, with progressive reduction of altered points at the 3rd month of treatment (SMS = 22), 6th month of treatment (SMS = 7), and 9th month of treatment (SMS = 3), culminating in complete restoration of tactile sensitivity by 12 months of treatment (SMS = 0). The sensitive mapping visually demonstrates the gradual replacement of impaired areas (marked by red-orange-violet) with green zones representing normal sensitivity (0.07 gf), thus corroborating the quantitative data from Table 7. Together, these findings support the utility of SMS as a sensitive and clinically meaningful tool for tracking neural recovery in HD neuropathy during treatment.

### 3.4 Correlations of SMS with neurological assessment

**FIGURE 2:**
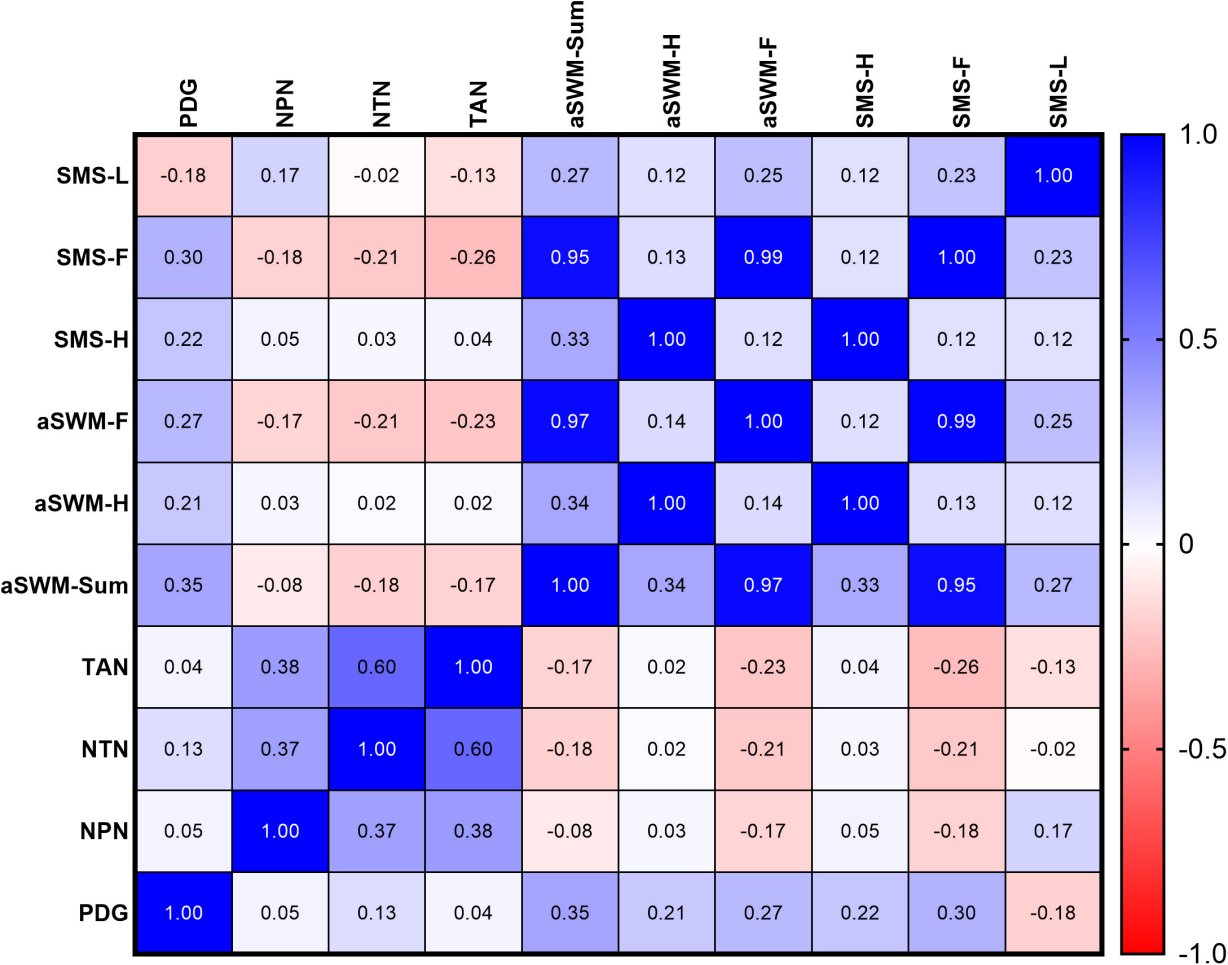
Spearman’s correlation analysis among the sensitive mapping score (SMS) of lesions, Semmes-Weinstein Monofilament (SWM) scores applied to hands and feet, number of altered nerves on palpation (pain and thickening), and Physical Disability Grade (PDG) at baseline. PDG, physical disability grade; NPN, Number of pained nerves on palpation; NTN, Number of thickened nerves on palpation; TAN, Total of Altered Nerves; aSWM, Sum of altered Semmes-Weinstein points; aSWM-H, Number of Semmes-Weinstein points altered on hands; aSWM-F, Number of Semmes-Weinstein points altered on feet; SMS-H, Sensitive Mapping Score applied on hands by SWM esthesiometry; SMS-F, Sensitive Mapping Score applied on feet by SWM esthesiometry; SMS-L, Sensitive Mapping Score on Lesion (average score by patients).

Data from Spearman’s correlation analyses among alterations in nerves, PDG, altered points in hand and feet esthesiometry, and application of SMS in hand and feet esthesiometry, and lesions at diagnosis are presented in Figure 2. The correlation matrix shows a positive correlation between Total of Altered Nerves (TAN) and Number of Pained Nerves on Palpation (NPN) [*r* = 0.38 (*p* = 0.016); weak]; NPN and Number of Thickened Nerves on Palpation (NTN) [*r* = 0.37 (*p* = 0.020); weak]; TAN and NTN [*r* = 0.60 (*p* < 0.0001); moderate]. The importance of esthesiometry in the assessment of neurological damage is evidenced by the positive correlation among Sum of Altered Semmes-Weinstein Points (aSWM-Sum) and Physical Disability Grade (PDG) [*r* = 0.35 (*p* = 0.025); weak]; Number of Semmes-Weinstein points altered on hands (aSWM-H) and aSWM-Sum [*r* = 0.34 (*p* = 0.031); weak]; Number of Semmes-Weinstein points altered on feet (aSWM-F) and aSWM-Sum [*r* = 0.97 (*p* < 0.0001); strong], highlighting the greater importance of the feet.

The analysis demonstrates validation of the SMS by evidencing a strong positive correlation with the esthesiometry findings: Sensitive Mapping Score applied on hands by SWM esthesiometry (SMS-H) and aSWM-H [*r* = 1.0 (*p* < 0.0001)]; Sensitive Mapping Score applied on feet by SWM esthesiometry (SMS-F) and aSWM-Sum [*r* = 0.95 (*p* < 0.0001)]; SMS-F and aSWM-F [*r* = 0.99 (*p* < 0.0001)]. Furthermore, the greater relevance of the sensitive mapping of the feet is supported by a positive correlation between PDG and SMS-F [*r* = 0.30 (*p* = 0.05)], as well as a weak correlation of SMS-H and aSWM-Sum [*r* = 0.33 (*p* = 0.036)], compared to the strong correlation observed with the feet.

## 4 Discussion

This study proposes the development of a SMS aimed at diagnosis, clinical follow-up, and the establishment of objective criteria for the cure of HD based on neurological recovery of tactile sensitivity. The investigation relies on systematic neurological evaluations of patients treated with the RIMOXCLAMIN regimen (21) and introduces a novel “sensitive mapping score” applied under the previously published mapping method to quantify sensory impairment in cutaneous lesions through standardized color-coded representations derived from SWM testing. As described by John and Govindharaj, the importance of neurological assessments focused on tactile sensitivity using the SWM, as well as the development of scoring systems for evaluating and monitoring leprosy cases, has been demonstrated (37).

Our findings reveal a significant discrepancy between the current operational classification, based solely on the number of skin lesions, and the broader clinical reality of HD. Neurological assessment showed tactile impairment in 95% of patients, with frequent alterations in thermal (65%) and pain sensitivity (60%). Yet, only 32.5% of the cohort presented with more than five cutaneous lesions, the threshold defining the MB classification. This confirms that current criteria may underestimate disease severity, potentially compromising therapeutic decisions and functional prognosis, as previously demonstrated by our group (26). Dermatologists specializing in HD have observed that clinically cured patients after MDT/WHO treatment can remain bacteriologically active, while bacteriologically negative patients can still show clinical activity (38). Patients presenting primarily neural symptoms often face challenges in establishing cure criteria, with many exhibiting clinical worsening and neural impairment even after treatment. There remains a knowledge gap regarding disease chronicity, as not all bacilli are eradicated by limited treatment regimens, resulting in persistent nerve function deficits (38).

Neurological manifestations frequently precede or occur independently of skin lesions (25, 26, 39–42). On nerve palpation, 80% of patients had thickened nerves, and 45% reported pain. Subjective complaints, including numbness (95%), tingling (80%), cramps (67.5%), weakness (65%), and neuropathic pain (75%) were highly prevalent. These data underscore the importance of detailed anamnesis and thorough physical examination to detect early neural involvement, even in clinically indeterminate cases. Santos et al. demonstrated that HD often begins with neurological symptoms before skin lesions appear, contributing to underdiagnosis and severe disabilities that perpetuate disease transmission (42).

Throughout treatment, a significant reduction in PDG was observed, with PDG 0 increasing from 15 to 77.5%, indicating substantial functional recovery. This aligns with findings by Frade et al. who reported superior PDG improvement with RIMOXCLAMIN compared to MDT/WHO, despite the RIMOXCLAMIN group started with a significantly higher proportion of patients classified as PDG2, which indicates a more severe baseline disability (21). Parallel improvements were documented in pain scores, nerve palpation findings, and neurological symptoms, reinforcing the internal consistency and clinical validity of our assessment tools.

Complementary exams demonstrated limited sensitivity when used individually. Only 30% of patients had positive laboratory test results. ELISA aPGL-I and RLEP-PCR showed positivity rates below 8 and 25%, respectively, while slit-skin smear was positive in 36.4% of the 27.5% of patients tested (43). In contrast, USG-PN and ENMG showed excellent diagnostic performance (100 and 91.9%, respectively), although their accessibility is limited by technical and logistical challenges (21, 34). Lima et al. reported a diagnostic sensitivity of 28.0% for aPGL-I in new HD cases, with 34.6% sensitivity and 96.0% specificity (35). A review of multiple studies found average sensitivities and specificities of 63.8 and 91%, respectively, mainly for MB cases (43). Additionally, qPCR may not detect patients with low bacillary loads and is often restricted to reference centers, limiting its field application (44). USG-PN offers a low-cost, non-invasive means of assessing nerves inaccessible to palpation, while nerve conduction studies yield approximately 88% sensitivity in HD diagnosis (2). A bidirectional review of medical records highlights a limitation in capturing laboratory tests at the time of diagnosis, reflecting the low proportion of individuals tested. Additionally, we found that patients with low bacillary loads but pronounced neurological signs and symptoms tend to show low seropositivity, especially in regions with lower endemicity.

Application of the SMS to hands and feet demonstrated gradual functional recovery. Improvements in the feet were evident from the third month and sustained thereafter. In the hands, simple counts of altered points reached significance only between baseline and month 6, whereas the SMS detected earlier improvements (*p* = 0.0156), confirming superior sensitivity to subtle neurological changes. This distinction may facilitate earlier interventions, particularly in mild or atypical clinical presentations.

Inspired by these findings, we developed and validated a lesion-specific score to standardize cutaneous sensory evaluation, replacing previous subjective assessments. At treatment completion, 74.32% of lesions showed normal sensitivity, with significant gains noted as early as the third (20.27%) and sixth (45.95%) months. Interestingly, lesion scores did not significantly correlate with peripheral nerve scores, suggesting that sensory impairment in skin lesions can occur independently of distal neuropathy. This supports the complementary role of lesion mapping in monitoring distinct but clinically relevant facets of neural function. Hypochromic macules with altered sensitivity are hallmark signs across the HD spectrum, and innovative lesion mapping provides valuable insights into the disease’s focality and asymmetry at diagnosis and during follow-up (1). In some cases, lesion recovery may even serve as an early biomarker of treatment response or failure, aiding tailored clinical management.

The concept of “administrative cure,” defined solely by completion of MDT within a fixed timeframe, warrants reconsideration in light of contemporary scientific understanding. WHO’s operational criteria were, at implementation, necessary for expanding treatment access in resource-limited, high-burden settings, standardizing care and ensuring treatment completion during a critical period. However, this pragmatic approach risks ossification into outdated dogma. Continuing to rely on rigid temporal cutoffs in 2025 disregards HD’s biological heterogeneity and the substantial fraction of patients with persistent neural deficits post-MDT. Declaring patients cured despite ongoing functional impairment is clinically insufficient and ethically problematic, as it fosters false assurance of disease resolution, delays relapse detection, and may perpetuate transmission.

Despite promising outcomes, this study has limitations, including a relatively small sample size *(n* = 40) and evaluation of a single treatment regimen (RIMOXCLAMIN). As also, conducting future studies that pair groups receiving different treatments to further clarify the effects on sensory improvement. Multicenter studies with larger, more diverse cohorts are needed to validate the SMS as a standardized marker of therapeutic efficacy and cure. Our findings underscore the inadequacy of time-based definitions. The SMS proposed here offers a reproducible, low-cost, and functionally meaningful alternative. By quantifying neurological recovery directly, SMS enables a patient-centered definition of cure anchored in objective clinical outcomes rather than bureaucratic endpoints. Moving toward a function-based criterion is no longer optional; it represents a necessary evolution in HD care that harmonizes scientific rigor with human dignity.

This study introduces and validates a novel SMS for assessing neurological recovery in HD, encompassing both distal nerve function and cutaneous sensory impairment. The structured application of SMS demonstrated strong responsiveness to clinical changes and outperformed traditional point-count methods in detecting early functional improvements. Improvements in lesion sensitivity scores paralleled reductions in neuropathic pain, nerve palpation abnormalities, and physical disability grades, underscoring the clinical relevance and internal consistency of the findings. Although correlations between domains were not strictly linear, the lesion score captured complementary aspects of neurological recovery, indicating its potential as a valuable marker of treatment response and disease progression. Long-term follow-up of patients with residual deficits post-treatment will be essential to discern patterns of spontaneous recovery, stability, or relapse, insights critical for shaping individualized therapy durations and managing refractory or resistant cases. Furthermore, evaluating how therapeutic adjustments influence SMS will enhance its utility in clinical decision-making.

In summary, the SMS offers a robust, accessible, and patient-centered tool to redefine clinical cure in HD. Its integration into routine practice promises to improve early detection of treatment failure, guide personalized therapy, and ensure that cure is defined not merely by elapsed time but by the meaningful restoration of function and patient dignity.

## Author’s note

Over the years, during clinical follow-up of patients with Hansen’s disease, I repeatedly observed a striking sensory dissociation between the center of skin lesions and the surrounding unaffected areas, a phenomenon historically described as the “island of anesthesia.” Despite its classic recognition, this pattern remained largely unquantified in clinical practice. Motivated by the need for improved documentation and visual monitoring, I began using color-coded, skin-safe pens to map tactile sensitivity across each lesion using Semmes-Weinstein Monofilaments (SWM). This approach provided an immediate visual representation of sensory impairment and its evolution throughout treatment. After several years of applying this method in routine practice, and publishing it as a series of medical images (1), the need for a standardized and reproducible system to quantify these color maps became evident. Through iterative clinical testing and refinement, a scoring system was developed to translate the qualitative mapping into a numerical scale. This scale aimed to capture the extent and severity of sensory impairment in a clinically meaningful and operationally simple way. The present study was designed to validate the Sensitive Mapping Score (SMS), evaluate its responsiveness during treatment, and assess its potential value as a tool for diagnosis, follow-up, and, ultimately, for redefining cure in Hansen’s disease in functional rather than merely temporal terms.

## Data Availability

The raw data supporting the conclusions of this article will be made available by the authors, without undue reservation.
